# Comparison of symptomatic spondylolysis in young soccer and baseball players

**DOI:** 10.1186/s13018-020-01910-4

**Published:** 2020-09-03

**Authors:** Takuji Yokoe, Takuya Tajima, Hiroshi Sugimura, Shinichirou Kubo, Shotarou Nozaki, Nami Yamaguchi, Yudai Morita, Etsuo Chosa

**Affiliations:** 1 Division of Orthopaedic Surgery, Department of Medicine of Sensory and Motor Organs, Faculty of Medicine, University of Miyazaki, 5200 Kihara, Kiyotake, Miyazaki, 889-1692 Japan; 2 Department of Radiology, Nozaki Higashi Hospital, 2105 Kouso, Murasumi, Miyazaki, 880-0837 Japan; 3 Department of Orthopaedic Surgery, Nozaki Higashi Hospital, 2105 Kouso, Murasumi, Miyazaki, 880-0837 Japan

**Keywords:** Spondylolysis, Magnetic resonance imaging, Soccer, Baseball

## Abstract

**Background:**

Spondylolysis is the main cause of low back pain (LBP) in young athletes. There are few studies analyzing the difference of spondylolysis among young athletes with different sports activities. The purpose of this study was to compare the clinical factors and distribution of the lesions of spondylolysis on magnetic resonance imaging (MRI) scans in young soccer and baseball players with symptomatic spondylolysis.

**Methods:**

The medical records of 267 young athletes aged 7 to 18 years old who underwent MRI to evaluate the cause of LBP between 2017 and 2020 were retrospectively reviewed to identify patients with spondylolysis. Of the young athletes with symptomatic spondylolysis, clinical factors and MRI findings in soccer and baseball players were retrospectively evaluated. The clinical factors were age, sex, interval from onset of LBP to MRI, and side of the dominant leg in the sports field. MRI findings included number, lumbar level, and side of the lesions.

**Results:**

A total of 33 soccer players (mean age, 15.4 ± 1.4 years) and 49 baseball players (mean age, 15.4 ± 1.6 years) with symptomatic spondylolysis were enrolled. All patients were male. No significant differences were noted in age and the interval from onset of LBP to MRI between the groups. Soccer players had greater numbers of multiple (*p* < 0.001) and bilateral (*p* < 0.001) lesions than baseball players. The dominant side of the hand for pitching or batting was correlated with the contralateral-side lesions in baseball players (*p* = 0.001).

**Conclusions:**

The distribution of the lesions of spondylolysis differed in young soccer and baseball players. Pitching or batting with the dominant-side hand would be associated with contralateral-side lesions in baseball players. Sports-specific movements and the side of the dominant leg should be considered when treating young athletes with symptomatic spondylolysis.

## Introduction

Sporting activity is regarded as an important predisposing factor in the development of spinal pathologies, and certain sports (e.g., weightlifting, wrestling, soccer, baseball, tennis, gymnastics) are considered risk factors [1–4]. The incidence of low back pain (LBP) in young athletes has been reported in several articles [2–5], and spondylolysis is a very common cause of LBP in young athletes [6]. One study reported that 47% of young athletes with LBP had spondylolysis [7]. Lumbar extensional and rotational movements, lumbar compression forces at landing, and the sacro-horizontal angle have been reported as important factors for developing lumbar spondylolysis [8, 9]. Spondylolysis occurs in individuals playing sports activities, and each sport requires specific movements and practice menu. It was reported that baseball and soccer were the greatest risk of spondylolysis in young male athletes [10]. Soccer demands several kinds of kicking, short sprints, collisions with other players, and occasionally throwing a ball with the hands. On the other hand, baseball requires mainly pitching and batting, and players use only the preferred side hand, although the dominant sides for pitching and batting are not always the same. Taking these different biomechanics required in each sport into account, it was hypothesized that the pathogenesis of spondylolysis would differ depending on the sport. However, to the best of our knowledge, there have been few studies reporting differences in the pathogenesis of spondylolysis depending on sports type.

Several studies demonstrated that magnetic resonance imaging (MRI) had a high diagnostic performance for spondylolysis and was useful for detecting early stage of spondylolysis [11, 12]. Short tau inversion recovery (STIR)-MRI is especially useful for accurate diagnosis of spondylolysis in both adult and adolescent athletes [13–16]. Therefore, evaluating lesions of spondylolysis using STIR-MRI would help clinicians to understand the characteristic pattern of spondylolysis in each sport. The purpose of this study was to evaluate the differences in clinical factors and MRI findings between young soccer and baseball players with symptomatic spondylolysis.

## Materials and methods

### Patients

This retrospective comparative study was approved by our institutional review board. The medical records of young athletes aged 7 to 18 years old who presented with LBP of undiagnosed etiology and underwent MRI scans to evaluate LBP in our hospital between January 1, 2017, and December 31, 2019, were reviewed to identify patients who were finally diagnosed with spondylolysis. Of these young athletes with symptomatic spondylolysis, young soccer and baseball players were retrospectively reviewed. All the included athletes had performed plain radiography of the lumbar spine prior to MRI scans. Plain radiography was performed in 4 viewings (anteroposterior, lateral, right, and left oblique viewing). Exclusion criteria were young athletes who were younger than 7 years and older than 18 years, had played soccer or baseball for less than 3 years at the time of first presentation to our hospital, and had a history of spondylolysis within 2 years from the MRI scan.

### Clinical factors and MRI findings

Clinical factors and MRI findings were compared between young soccer and baseball players with symptomatic spondylolysis. Clinical factors and patient characteristics (sex, age, side of the dominant leg in the sports activity, and interval from onset of the LBP to MRI scans) were evaluated by means of a chart review. Information regarding the side of the dominant leg in the sports activity was prospectively collected by telephone when medical records did not contain the information. MRI findings included the number of lesions, the lumbar levels of the lesions, and the side of the lesions. Multiple lesions were defined as lesions distributed at more than 2 pars interarticularises. Multilevel lesions were defined as lesions distributed at more than 2 levels of the lumbar spine. Bilateral lesions were defined as lesions distributed at bilateral sides of the lumbar spine. The correlation between the side of the dominant leg and the side of the lesion was further assessed. The lesion of spondylolysis was defined as a high signal intensity lesion at the pars interarticularis on STIR-MRI scans [14, 16]. MRI scans were evaluated by an experienced radiologist who was not involved in both treatment of the included patients and collection of the medical records.

All MRI scans were performed with the patients in the supine position with extended legs and without the use of general anesthesia or contrast enhancement. Subjects were examined using a research-dedicated 1.5 Tesla whole-body MRI scanner (Vantage, Canon Medical Systems, Ohtawara, Japan) with a standard spine coil. T2-weighted fast spin-echo scans with spectral adiabatic inversion recovery fat suppression technique were taken with TE of 60 ms, TR of 4000–6000 ms, a flip angle of 90°, slice thickness/gap 4.0/0.8 mm, field of view 30 × 30 cm^2^, and matrix size 256 × 256.

### Statistical analysis

To compare the two sports groups, chi-squared test or Fisher’s exact test for categorical variables and the Mann-Whitney *U* test for continuous variables were performed. All statistical analyses were performed using SPSS software (version 21.0, SPSS, Chicago, IL), and the significance of the threshold was set at *p* < 0.05.

## Results

### Patient demographics and clinical factors

**Fig. 1:**
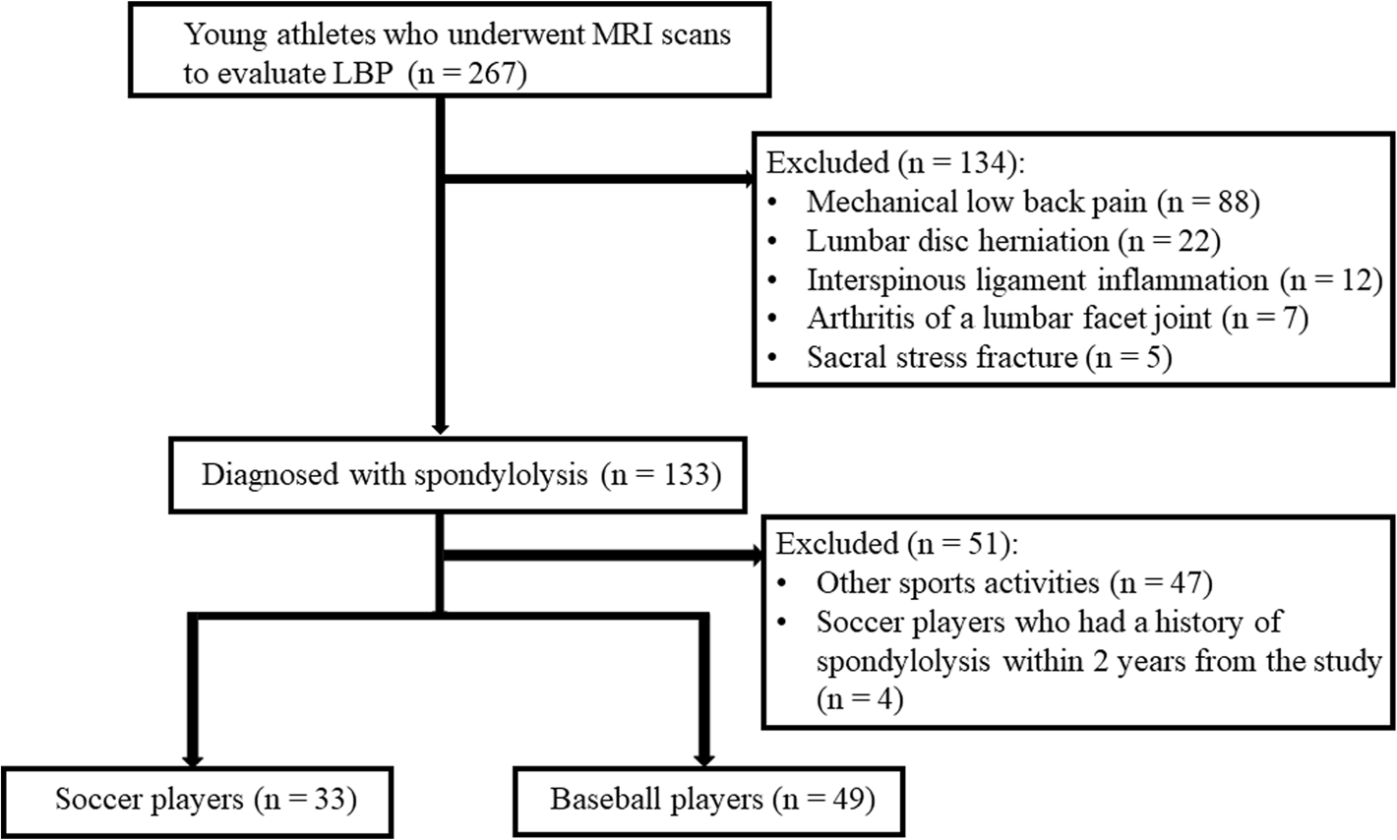
Flow chart for patient enrolment. MRI, magnetic resonance imaging; LBP, low back pain

A total of 267 young athletes who underwent MRI scans to evaluate LBP were identified, and 133 patients were finally diagnosed with spondylolysis. Of the 133 young athletes with symptomatic spondylolysis, 47 were excluded for participating in a sports activity other than soccer and baseball, and 4 were excluded for a history of spondylolysis within 2 years from their MRI scans. Therefore, 33 soccer players and 49 baseball players were enrolled in this study (Fig. 1). All the included athletes had extension-related LBP, and there were no radiographic findings suggesting the presence of spondylolysis such as defects or sclerosing of the pars interarticularis. The details of both groups are shown in Table 1. All patients were male in both groups. The mean age of the soccer players and baseball players was 15.4 ± 1.4 and 15.4 ± 1.6 years, respectively (n.s.). The mean interval from onset of LBP to MRI scans in soccer players and baseball players was 3.5 ± 2.4 and 4.3 ± 4.1 weeks, respectively (n.s.).

**Table 1:** Details of soccer players and baseball players with symptomatic spondylolysis

	Soccer players	Baseball players	*p* value
Number	33	49	
Age (years)	15.4 ± 1.4	15.4 ± 1.6	n.s.
Interval from onset of low back pain to MRI (weeks)	3.5 ± 2.4	4.3 ± 4.1	n.s.
Number (%) of players with multiple HSL on STIR-MRI, *n* (%)	21 (63.6%)	10 (20.4%)	< 0.001
Number (%) of players with HSL at multiple lumbar levels on STIR-MRI, *n* (%)	7 (21.2%)	2 (4.1%)	0.03
Number (%) of players with bilateral HSL on STIR-MRI, *n* (%)	20 (60.6%)	9 (18.4%)	< 0.001

Data are expressed as mean ± SD unless otherwise indicated

*HSL* high signal lesion, *STIR* short tau inversion recovery, *MRI* magnetic resonance imaging, *n.s.* not significant

### MRI findings

A total of 56 lesions were detected in 33 soccer players, and 60 lesions were detected in 49 baseball players. Spondylolysis located at L5 was identified in 55.4% (31/56) of soccer players and 60.0% (36/60) of baseball players, followed by L4 in 30.4% (17/56) of soccer players and 33.3% (20/60) of baseball players (Table 2). Soccer players with spondylolysis, compared with baseball players, had a greater number of multiple lesions (63.6% vs 20.4%, *p* < 0.001), multiple lumbar level lesions (21.2% vs 4.1%, *p* = 0.03), and bilateral lesions (60.6% vs 18.4%, *p* < 0.001) (Table 1).

**Table 2:** MRI findings in soccer players and baseball players: sites of high signal lesions at pars interarticularis

Lumbar level	Sites of the lesions at pars interarticularis						
	Soccer players (*n* = 33)			Baseball players (*n* = 49)			Soccer players and baseball players (*n* = 82)
	Right	Left	Subtotal	Right	Left	Subtotal	Total
L3	3	5	8	1	3	4	12
L4	9	8	17	7	13	20	37
L5	15	16	31	11	25	36	67
Total	27	29	56	19	41	60	116

### Correlation between the side of the dominant leg and side of the lesion

Table 3 summarizes the side of the dominant leg in the sports activity and the side of the lesions. In soccer players, the side of the dominant foot was right in 87.9% (group S). The side of the lesions of group S was bilateral in 65.5%. In baseball players, players whose dominant hand was right for both pitching and batting accounted for 67.3% (group A), players whose dominant hand for pitching was right and for batting was left accounted for 26.5% (group B), and players whose dominant hand was left for both pitching and batting accounted for 6.1% (group C). In group A, the side of the lesion was left in 69.7%, followed by bilateral in 18.2%. In group B, the side of the lesion was left in 46.2%, followed by right in 30.8%. In group C, all lesions were on the right. The results of a comparison of the side of the lesions and the side of the dominant leg among group S, group A, and group B are shown in Table 4. There was a statistically significant difference in left-sided lesions (*p* = 0.001) and bilateral lesions (*p* < 0.001) among the three groups. Group A had a greater number of left-sided lesions compared with group S (69.7% vs 24.1%, *p* < 0.001). Group S had a greater number of bilateral lesions compared with group A (65.5% vs 18.2%, *p* < 0.001) and group B (65.5% vs 23.1%, *p* = 0.02).

**Table 3:** Side of the dominant leg and side of the lesions on STIR-MRI in soccer players and baseball players

	Right	Left	Bilateral
Soccer players (*n* = 33)			
Side of the dominant foot			
Right (*n* = 29)	3 (10.3%)	7 (24.1%)	19 (65.5%)
Left (*n* = 4)	2 (50.0%)	1 (25.0%)	1 (25.0%)
Total	5 (15.2%)	8 (24.2%)	20 (60.6%)
Baseball players (*n* = 49)			
Side of the dominant hand (pitching/batting)			
Right/right (*n* = 33)	4 (12.1%)	23 (69.7%)	6 (18.2%)
Right/left (*n* = 13)	4 (30.8%)	6 (46.2%)	3 (23.1%)
Left/left (*n* = 3)	3 (100%)	0 (0%)	0 (0%)
Total	11 (22.4%)	29 (59.2%)	9 (18.4%)

Data are expressed as number (%) unless otherwise indicated

*STIR* short tau inversion recovery, *MRI* magnetic resonance imaging

**Table 4:** Correlation between the side of the dominant leg and side of the lesions in soccer and baseball players

	Group S (*n* = 29)	Group A (*n* = 33)	Group B (*n* = 13)	*p* value			
				All groups	Group S vs group A	Group S vs group B	Group A vs group B
Side of the lesions on STIR-MRI							
Right	3 (10.3%)	4 (12.1%)	4 (30.8%)	n.s.	n.s.	n.s.	n.s.
Left	7 (24.1%)	23 (69.7%)	6 (46.2%)	0.001	< 0.001	n.s.	n.s.
Bilateral	19 (65.5%)	6 (18.2%)	3 (23.1%)	< 0.001	< 0.001	0.02	n.s.

Data are expressed as number (%) unless otherwise indicated

Group S, soccer players with the right side of the dominant foot

Group A, baseball players whose both dominant sides of the hand of pitching and batting were right

Group B, baseball players whose dominant side of the hand of pitching was right and that of batting was left

*STIR* short tau inversion recovery, *MRI* magnetic resonance imaging, *n.s.* not significant

## Discussion

The most important finding of this study was that the distribution of the lesions of spondylolysis on MRI differed between young soccer and baseball players, which suggested different pathomechanisms of spondylolysis in each sports activity. This was the first study, to the best of our knowledge, that investigated the differences in clinical factors and MRI findings between young soccer and baseball players with symptomatic spondylolysis. In fact, most previous studies have reported the results of patients with spondylolysis in individual sports [17–19], and only one study compared spondylolysis in cricketers and soccer players using single-photon emission computerized tomography (SPECT) [20]. In the present study, soccer players with spondylolysis had more multiple and bilateral lesions compared with baseball players (*p* < 0.001). Baseball players with spondylolysis tended to have a single, unilateral lesion compared with soccer players, and pitching or batting with the dominant hand was likely to affect the contralateral side of the pars interarticularis. The symmetrical distribution of lesions of spondylolysis in soccer players was consistent with results previously described by Gregory et al., although the lesions were assessed by SPECT [20].

It was speculated that the greater numbers of multiple and symmetrical lesions of spondylolysis in soccer players were due to specific movements and tendencies required when playing soccer. Most soccer players mainly use the dominant foot, but often also use the non-dominant foot to kick a ball. Moreover, during training and matches, in general, they spend more than 95% of their time jogging and running quickly without kicking a ball. On the other hand, baseball players routinely generate high rotational and torsional forces on the lumbar spine during pitching and batting. Pitching particularly can lead to back stiffness and facet joint of discogenic pain, and swinging a bat generates high compressive loads on the spine [21]. They use only the dominant hand when pitching and batting, and they tend to repeat the same movements. These specific movements would lead to lesions of spondylolysis located contralateral to the dominant side of the hand for pitching or batting in baseball players. One of the strong points in this study was that information regarding the dominant leg in the sports field was collected prospectively in order to describe the location of the lesions relative to the dominant side of the leg in soccer and baseball. This was the first report, as far as we know, describing the correlation between the side of the lesions and the side of the dominant leg in soccer and baseball players with spondylolysis.

Lesions of spondylolysis were located at lower lumbar levels, 57.8% at L5, 31.9% at L4, and 10.3% at L3, with no lesions at L1 and L2 in the present study. Ladenhauf et al. reported that the levels of spondylolysis for 127 young athletes were L5 in 74%, L4 in 22.1%, and L3 in 1.6%, although they evaluated lesions of spondylolysis using various kinds of imaging modalities including MRI, SPECT, CT, and X-ray [22], and the present results showed a similar lesion distribution. The most affected lumbar level was L5 in both groups in the present study; 55.4% of soccer players and 60.0% of baseball players revealed high signal intensity lesions at L5 on STIR-MRI scans. Congeni et al. reported that 71% of spondylolysis was seen at L5 [23], and Gregory et al. reported 66.7% [20], and the present results were consistent with these results.

The risk of pediatric and adolescent sports injuries was high, and children 5–14 years accounted for nearly 40% of all sports-related injuries [24]. Spondylolysis is regarded as the main cause of LBP in young athletes [6]. Young athletes with spondylolysis are mainly treated conservatively with the cessation of sports activity and rehabilitation with or without a thoracolumbar orthosis, with favorable clinical outcomes [25–27]. It was reported that the mean time of cessation of sports activity was 3.9 ± 0.8 months, with a mean time of 5.2 ± 2.1 months for a complete return to soccer in 34 young soccer players with spondylolysis who were treated nonoperatively [28]. Lumbar flexion-extension movements, rotational forces of the trunk, and lumbar compression forces at landing have been reported as predisposing factors to spondylolysis [8, 9]. Based on the results of the present study, the pathomechanism of spondylolysis would differ between young soccer and baseball players with spondylolysis, and in baseball players, the dominant-side hand for pitching and batting would affect the contralateral side of the pars interarticularis. Therefore, clinicians should consider not only previously reported predisposing factors for spondylolysis, but also characteristic movements and training patterns in each sport and the side of the dominant leg in the sports field when determining the regimen of conservative treatments for young athletes with spondylolysis. For example, when we perform the strengthening of core abdominal muscles, which is an important conservative treatment for spondylolysis that can stabilize pars defects [29, 30], for baseball players with spondylolysis whose dominant-side hand for pitching is the right side, the left side of the core abdominal muscles should be strengthened more. For young soccer players with spondylolysis, we should consider symmetrical strengthening of abdominal muscles and resistance exercises of back extension and rotary torso. To clarify the efficacy of such individualized conservative treatments for athletes with spondylolysis, prospective studies will be needed in the future.

This study had several limitations. First, the sample size was small in each group. Second, all the young athletes included in this study were male; therefore, spondylolysis was not evaluated in young female soccer and baseball players. Third, young soccer and baseball players with spondylolysis who did not undergo MRI, including players with asymptomatic spondylolysis, were not included, which allows for selection bias. Fourth, CT and SPECT were not used for the assessment of the lesions of spondylolysis. Some authors reported that MRI was not as sensitive for detecting spondylolysis as a SPECT bone scan [31, 32]; however, it was confirmed that MRI has a high diagnostic performance for detecting a pars defect in young athletes [11, 12, 14, 16, 33]. MRI would be preferable for young athletes because of the lack of ionizing radiation. Fifth, young athletes with spondylolysis who participated in a sports activity other than soccer and baseball were not evaluated. Further case-controlled studies may be needed to clarify whether there are specific findings of spondylolysis depending on the sports type. Finally, findings of physical examinations, such as tightness of the hamstrings and hip range of motion, were not assessed due to the retrospective study design. Despite these limitations, the present study provided important information for understanding the pathomechanism of spondylolysis in young soccer and baseball players.

## Conclusions

Young soccer players with symptomatic spondylolysis had more multiple and bilateral lesions than baseball players with symptomatic spondylolysis. In baseball players, pitching or batting with the dominant hand was associated with lesions located at the contralateral side of the pars interarticularis. Clinicians should consider sports-specific movements and the side of the dominant leg in the sports activity when creating individualized regimens of conservative treatments for young athletes with symptomatic spondylolysis.

## Abbreviations

LBP: Low back pain
MRI: Magnetic resonance imaging
STIR: Short tau inversion recovery
CT: Computer tomography
SPECT: Single-photon emission computerized tomography

## References

[1] Maffulli N, Longo UG, Gougoulias N, Caine D, Denaro V (2011). Sport injuries: a review of outcomes. Br Med Bull.

[2] Rossi F, Dragoni S (1990). Lumbar spondylolysis: occurrence in competitive athletes. Updated achievements in a series of 390 cases. J Sports Med Phys Fitness.

[3] Gurd DP (2011). Back pain in the young athlete. Sports Med Arthrosc Rev.

[4] Kujala UM, Taimela S, Erkintalo M, Salminen JJ, Kaprio J (1996). Low-back pain in adolescent athletes. Med Sci Sports Exerc.

[5] Muller J, Muller S, Stoll J, Frohlich K, Otto C, Mayer F (2017). Back pain prevalence in adolescent athletes. Scand J Med Sci Sports.

[6] Garry JP, McShane J (1998). Lumbar spondylolysis in adolescent athletes. J Fam Pract.

[7] Micheli LJ, Wood R (1995). Back pain in young athletes. Arch Pediatr Adolesc Med.

[8] Farfan HF, Osteria V, Lamy C (1976). The mechanical etiology of spondylolysis and spondylolisthesis. Clin Orthop Relat Res.

[9] Sward L, Hellstrom M, Jacobsson B, Peterson L (1989). Spondylolysis and the sacro-horizontal angle in athletes. Acta Radiol.

[10] Selhorst M, Fischer A, MacDonald J (2019). Prevalence of spondylolysis in symptomatic adolescent athletes: an assessment of sport risk in nonelite athletes. Clin J Sport Med.

[11] Dhouib A, Tabard-Fougere A, Hanquinet S, Dayer R (2018). Diagnostic accuracy of MR imaging for direct visualization of lumbar pars defect in children and young adults: a systematic review and meta-analysis. Eur Spine J.

[12] Rush JK, Astur N, Scott S, Kelly DM, Sawyer JR, Warner WC (2015). Use of magnetic resonance imaging in the evaluation of spondylolysis. J Pediatr Orthop.

[13] Sairyo K, Sakai T, Mase Y, Kon T, Shibuya I, Kanamori Y, Kosugi T, Dezawa A (2011). Painful lumbar spondylolysis among pediatric sports players: a pilot MRI study. Arch Orthop Trauma Surg.

[14] Ganiyusufoglu AK, Onat L, Karatoprak O, Enercan M, Hamzaoglu A. Diagnostic accuracy of magnetic resonance imaging versus computed tomography in stress fractures of the lumbar spine. 2010;65:902-907. 10.1016/j.crad.2010.06.011 20933645

[15] Yamashita K, Sugiura K, Manabe H, Ishihama Y, Tezuka F, Takata Y, Sakai T, Maeda T, Sairyo K (2019). Accurate diagnosis of low back pain in adult elite athletes. J Med Investig.

[16] Cheung KK, Dhawan RT, Wilson LF, Peirce NS, Rajeswaran G (2018). Pars interarticularis injury in elite athletes – the role of imaging in diagnosis and management. Eur J Radiol.

[17] Iwamoto J, Abe H, Tsukimura Y, Wakano K (2004). Relationship between radiographic abnormalities of lumbar spine and incidence of low back pain in high school and college football players: a prospective study. Am J Sports Med.

[18] Jackson DW, Wiltse LL, Cirincoine RJ (1976). Spondylolysis in the female gymnast. Clin Orthop Relat Res.

[19] Ogon M, Riedl-Huter C, Sterzinger W, Krismer M, Spratt KF, Wimmer C (2001). Radiographic abnormalities and low back pain in elite skiers. Clin Orthop Relat Res.

[20] Gregory PL, Batt ME, Kerslake RW (2004). Comparing spondylolysis in cricketers and soccer players. Br J Sports Med.

[21] Dines JS, Altcheck DW, Andrews JR. Sports medicine of baseball. Wolter Kluwer Health/Lippincott Williams and Wilkins Lumbar injuries. 2012:383–98.

[22] Laudenhauf HN, Fabricant PD, Grossman E (2013). Athletic participation in children with symptomatic spondylolysis in the New York area. Med Sci Sports Exerc.

[23] Congeni J, McCulloch J, Swanson K (1997). Lumbar spondylolysis. A study of natural progression in athletes. Am J Sports Med.

[24] Maffulli N, Caine D (2005). The epidemiology of children’s team sports injuries. Med Sports Sci.

[25] Blanda J, Bethem D, Moats W, Lew M (1993). Defects of pars interarticularis in athletes: protocol for nonoperative treatment. J Spinal Disord.

[26] El Rassi G, Takemitsu M, Woratanarat P, Shah SA (2005). Lumbar spondylolysis in pediatric and adolescent soccer players. Am J Sports Med.

[27] Jackson DW, Wiltse LL, Dingeman RD, Hayes M (1981). Stress reaction involving the pars interarticularis in young athletes. Am J Sports Med.

[28] Alvarez-Díaz P, Alentorn-Geli E, Steinbacher G, Rius M, Pellisé F, Cugat R (2011). Conservative treatment of lumbar spondylolysis in young soccer players. Knee Surg Sports Traumatol Arthrosc.

[29] Stuber KJ, Bruno P, Sajko S, Hayden JA (2014). Core stability exercises for low back pain in athletes: a systematic review of the literature. Clin J Sport Med.

[30] You JH, Kim SY, Oh DW, Chon SC (2014). The effect of a novel core stabilization technique on managing patients with chronic low back pain: a randomized, controlled, experimenter-blinded study. Clin Rehabil.

[31] Masci L, Pike J, Malara F, Phillips B, Bennell K, Brukner P (2006). Use of the onelegged hyperextension test and magnetic resonance imaging in the diagnosis of active spondylolysis. Br J Sports Med.

[32] Zetaruk M, Micheli LJ, Purcell LK et al. Lumbar spine injuries. In: The adolescent athlete. Springer, New York. 2007:109-140.

[33] Kobayashi A, Kobayashi T, Kato K, Higuchi H, Takagishi K (2013). Diagnosis of rediographically occult lumbar spondylolysis in young athletes by magnetic resonance imaging. Am J Sports Med.

